# Supplementary material for: High temperature limit of photosynthetic excitons
Source: Nat Commun. 2018 Jan 8;9:99. doi: 10.1038/s41467-017-02544-7 (PMC5758513; doi:10.1038/s41467-017-02544-7)
Supplement: Supplementary file 1 — Supplementary Figures [file 41467_2017_2544_MOESM1_ESM.pdf]

Supplementary Figures

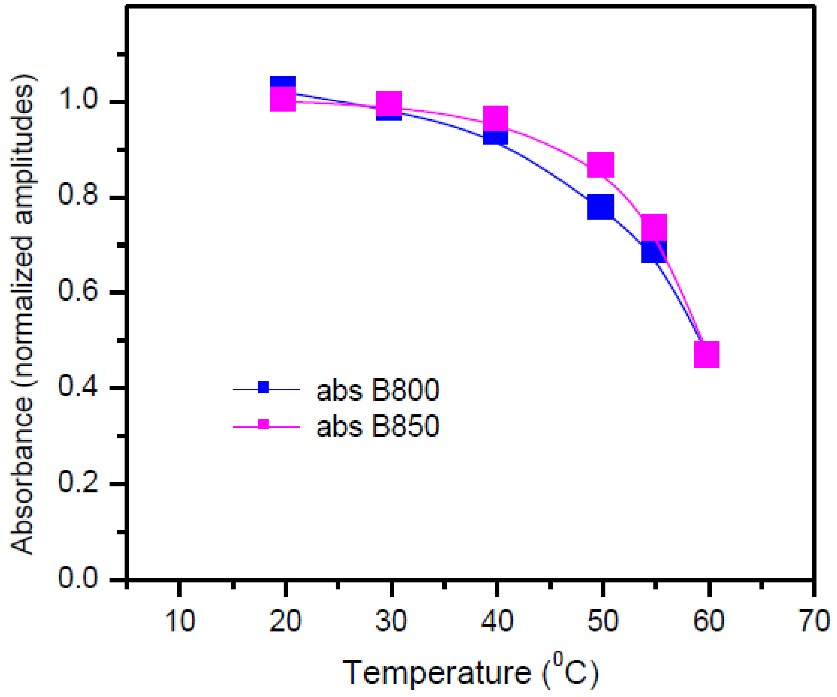

Supplementary Figure 1 | Decrease of absorbance

Temperature dependences of normalized B800 and B850 absorbance peaks of the isolated LH2 complex from *Rhodoblastus* (*R.) acidophilus*.

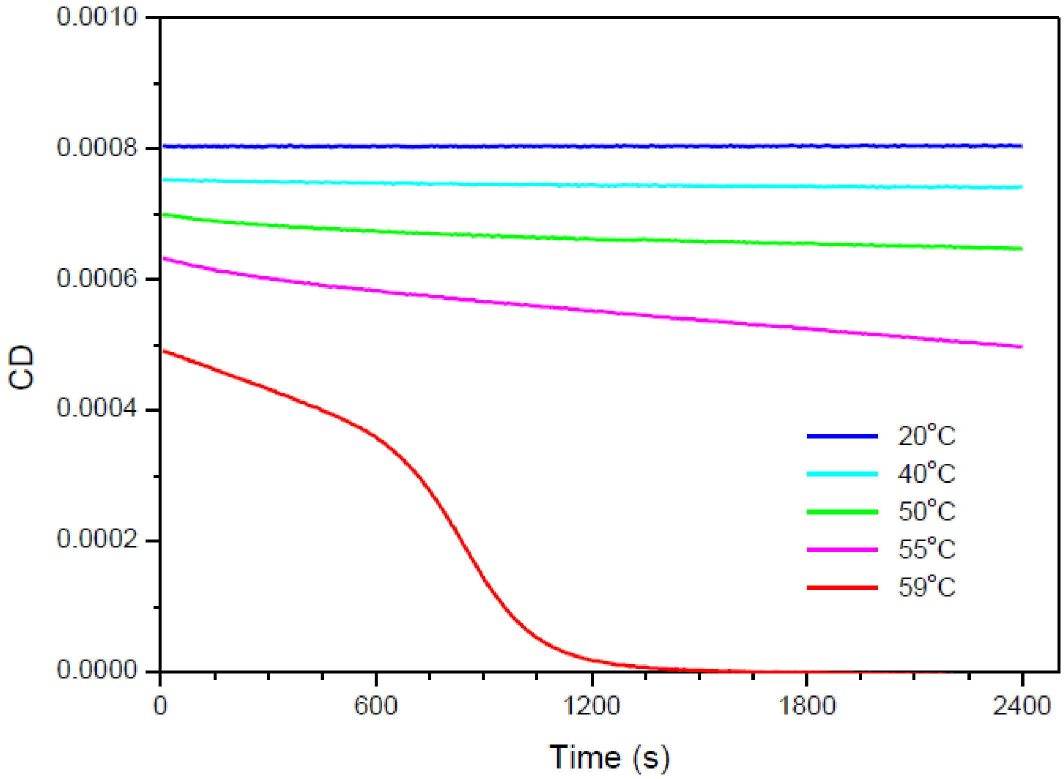

Supplementary Figure 2 | Stability of sample at selected temperatures

Time dependence of circular dichroism (CD) intensity of the isolated LH2 complex from *R. acidophilus* at the carotenoid 531 nm line recorded at temperatures indicated. Optical density of sample was 0.72 at 858 nm in a 1 mm cuvette. For every measurement a fresh sample was prepared, which was rapidly heated to the selected temperature.

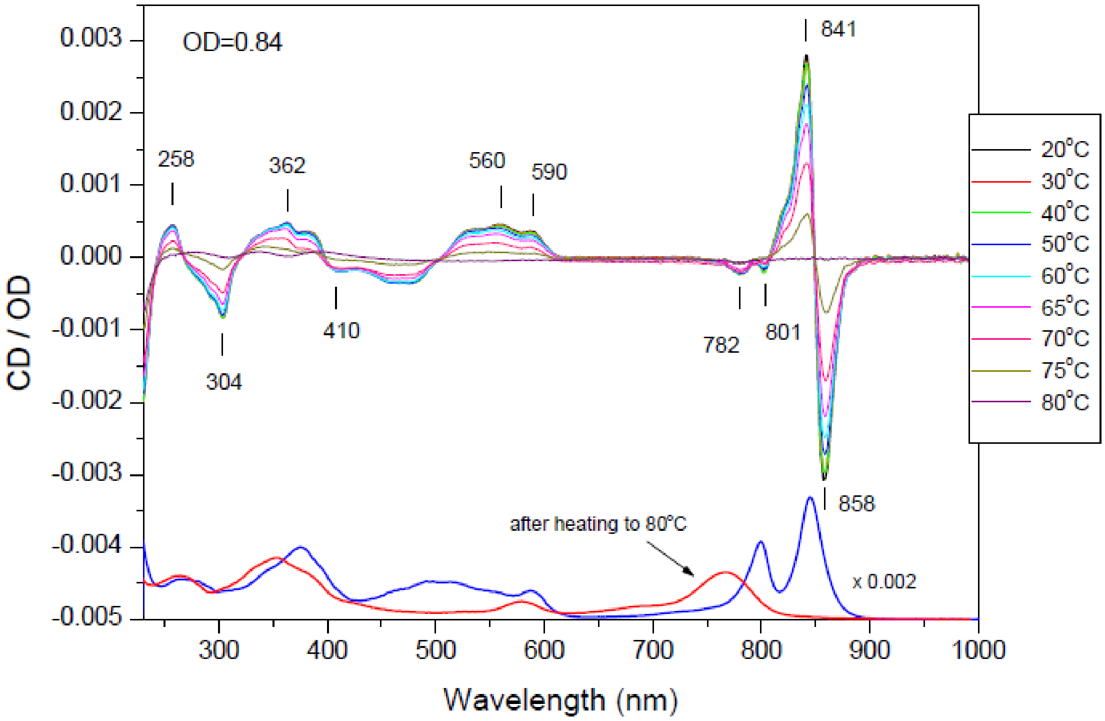

Supplementary Figure 3 | CD spectra of isolated LH2 complex from *Rba. sphaeroides*

Normalized to optical density CD spectra of the isolated LH2 complex from *Rhodobacter* (*Rba.) sphaeroides* at different temperatures. Numbers at corresponding vertical lines indicate the positions of the CD spectral lines in nanometers. These lines were selected for presentation in Supplementary Fig. 3. The 20°C absorption spectrum (blue line) with optical density (OD) of 0.84 at 845 nm was multiplied by a factor of 2×10^{-3} and shifted vertically to show absorption and CD spectra in one figure. Shown by red line is the absorption spectrum measured at 20°C after heating up the sample to 80°C and cooling it down.

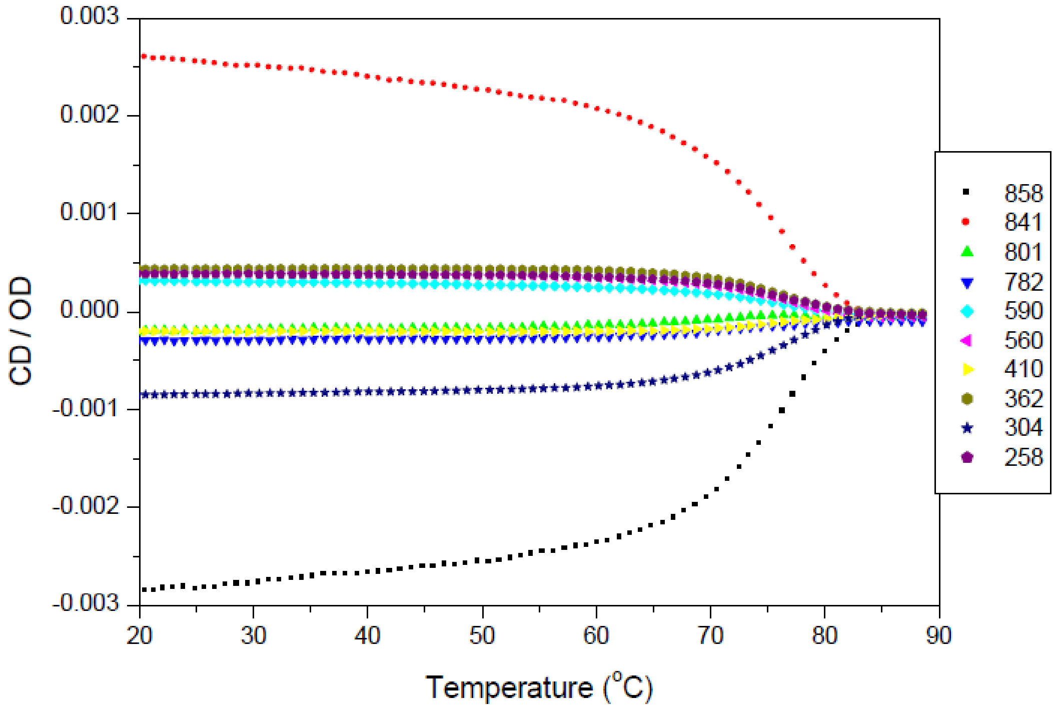

Supplementary Figure 4 | Temperature dependence of CD lines in isolated *Rba. sphaeroides*

Temperature dependence of the intensities of the selected CD spectral lines (indicated in nanometers) of the isolated LH2 complex from *Rba. sphaeroides*. The sample temperature was raised with a rate of 1°C/min; measurement time per temperature point was ~1 min.

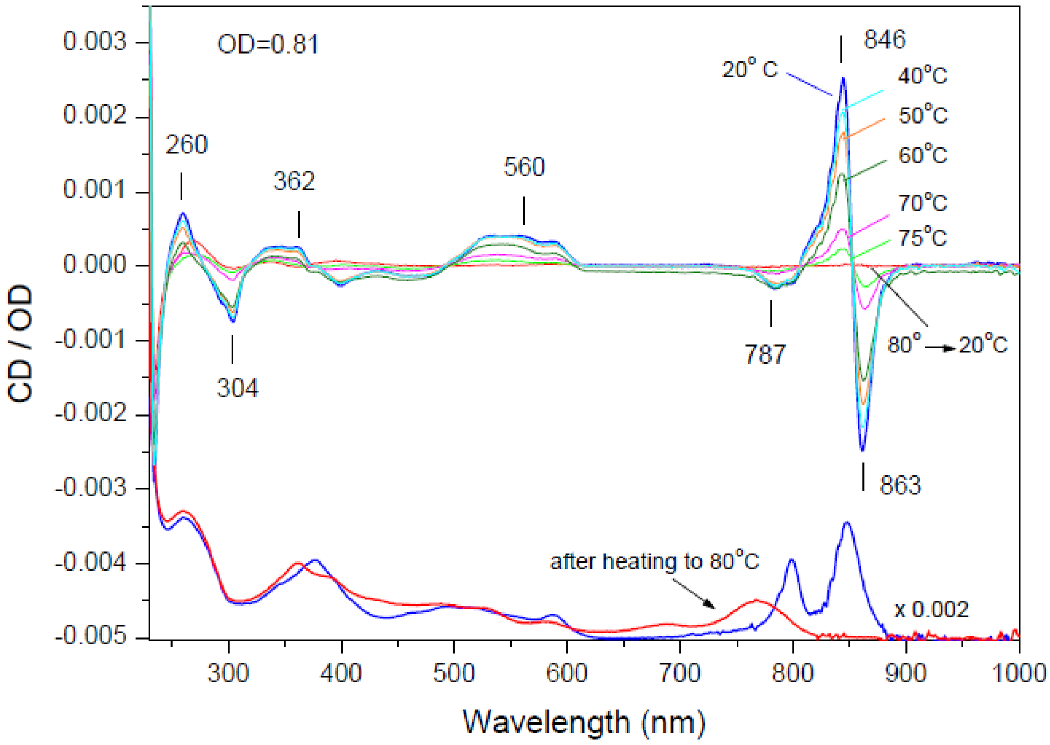

Supplementary Figure 5 | CD spectra of membrane LH2 complex from *Rba. sphaeroides*

Normalized to optical density CD spectra of the membrane LH2 complex from *Rba. sphaeroides* at different temperatures. Numbers at corresponding vertical lines indicate the positions of the CD spectral lines in nanometers. These lines were selected for presentation in Supplementary Fig. 5. The 20°C absorption spectrum (blue line) with optical density (OD) of 0.81 at 847 nm was multiplied by a factor of $2{\times}10^{-3}$ and shifted vertically to show absorption and CD spectra in one figure. Shown by red line is the absorption spectrum measured at 20°C after heating up the sample to 80°C and cooling it down.

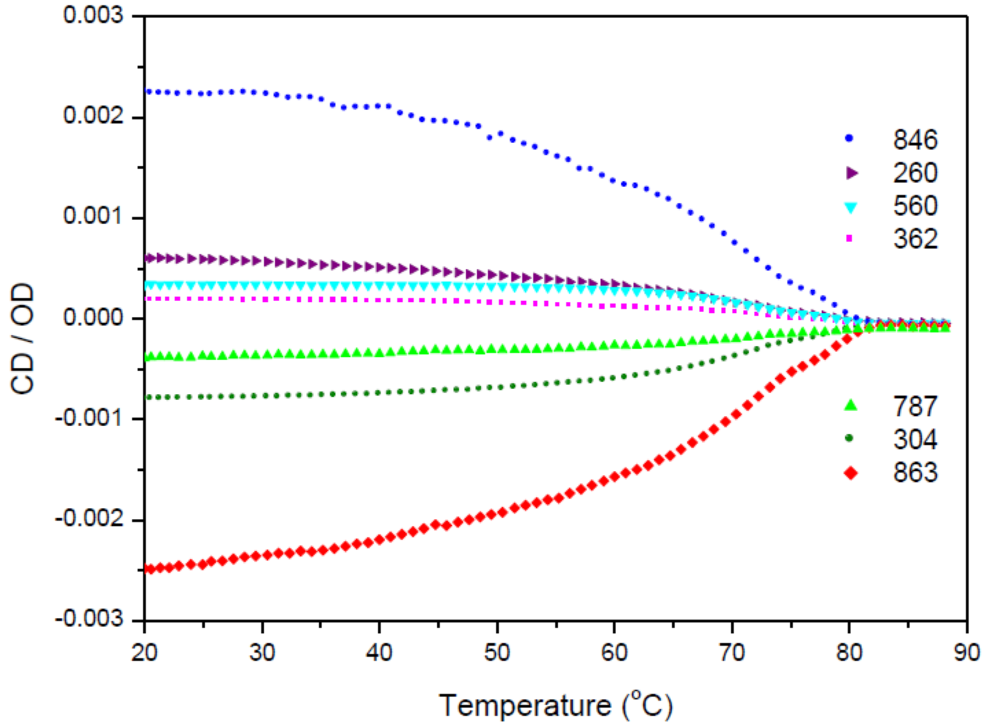

Supplementary Figure 6 | Temperature dependence of CD lines in membrane *Rba. sphaeroides*

Temperature dependence of the intensities of the selected CD spectral lines (indicated in nanometers) of the membrane LH2 complex from *Rba. sphaeroides*. The sample temperature was raised with a rate of 1°C/min; measurement time per temperature point was ~1 min.
